# Alpha-gal sensitization and allergic transfusion reactions: a scoping review protocol

**DOI:** 10.1186/s13643-026-03134-9

**Published:** 2026-02-24

**Authors:** Maureen J. Miller, Carrie Price, Tracy C. Shields, Roya Zarpak, Patricia Lee, Zachary Osterwind, Mattias Lenz, Mirna Argueta Guevara, Sarah Fowler, Alicia A. Livinski, Valeria De Giorgi

**Affiliations:** 1 Department of Transfusion Medicine, NIH Clinical Center, 10 Center Drive, Bethesda, MD USA; 2 NIH Library, Office of Research Services, Office of the Director, National Institutes of Health, Bethesda, MD USA

**Keywords:** Blood donor testing, Alpha-gal (α-Gal) syndrome, Alpha-gal (α-Gal) sensitization, Tickborne illnesses, Transfusion-transmitted infections, Scoping review

## Abstract

**Background:**

Tick bites may expose individuals to a carbohydrate not found in humans, galactose-alpha-1,3-galactose (alpha-gal). A spectrum of disorders may result from IgE-mediated hypersensitivity reactions to alpha-gal, including alpha-gal syndrome (AGS)*,* an allergy to meat or meat-derived products usually presenting 2–6 h after consuming the product plus positive alpha-gal specific IgE testing for the oligosaccharide. Reports of anaphylaxis in group O recipients of group B plasma in the absence of other risk factors for severe allergic reactions to blood transfusion could be alpha-gal sensitization; the allergen galactose-alpha-1,3-galactose (Gal-alpha-1-3Galβ1-(3)4GlcNAc-R) is antigenically similar to the B blood group antigen (Gal-alpha-1–3(Fuc-alpha-1,2)Gal)*.* The potential cross-reactivity of alpha-gal specific IgE to B type red blood cells may pose a safety consideration for blood donation and transfusion. This scoping review protocol will be used to research all publications on alpha-gal sensitization to (1) describe characteristics of all known cases of transfusion-related alpha-Gal syndrome (TRAGS) and hypersensitivity reactions to infusions of mammalian red meat-derived medical products besides blood components that may resemble TRAGS; (2) identify studies that explore possible relationships between alpha-gal sensitization and blood group that may be relevant to understanding TRAGS; (3) describe which clinical, laboratory, and epidemiologic parameters used to diagnose AGS food allergy are also appropriate to diagnose TRAGS; and (4) identify which diagnostic assays exist for AGS and how they are used for AGS and/or TRAGS.

**Methods:**

Using peer-reviewed search strategies, our study team will perform a scoping review with no date or language limit of all literature relevant to the research objectives in PubMed, Scopus, Embase, Web of Science, and Cochrane Central Register of Controlled Trials, including title, abstract, full-text screening, and data collection using Covidence.

**Discussion:**

This study involves published data predominantly from humans (and, rarely, animal) studies of diagnostic assays in development for use in humans. It does not require institutional review board or ethics approval. We intend to disseminate our findings to specialists in allergy, immunology, hematology, and blood banking and to patients or blood donors experiencing symptoms of alpha-gal sensitization.

**Systematic review registration:**

Open Science Framework (osf.io) (DOI: 10.17605/OSF.IO/WDZT6).

**Supplementary Information:**

The online version contains supplementary material available at 10.1186/s13643-026-03134-9.

## Background

Tick bites from species such as the lone star tick (*Amblyomma americanum*) endemic to the southeastern United States expose individuals to a carbohydrate not found in humans, galactose-alpha-1,3-galactose (alpha-gal). A spectrum of symptoms may result from IgE-mediated hypersensitivity reactions to exposure to alpha-gal, known as alpha-gal syndrome (AGS)*.* AGS is a diagnosis involving an allergy (e.g., unexplained hives, swelling, or other new allergic reactions that may induce severe, life-threatening anaphylaxis) to meat or meat-derived products usually presenting 2–6 h after consuming the product and positive alpha-gal specific IgE testing for the oligosaccharide. Thousands of people in the USA may be affected [1–3].

AGS-like symptoms have also been observed in people without known exposure to meat or meat products. Multiple risk factors that may lead to sensitization in these individuals have been explored, most recently blood transfusions. Recent reports of anaphylaxis in group O recipients of group B plasma from allogeneic blood products (healthy volunteer donors) in the absence of other risk factors for severe allergic reactions to blood transfusion (e.g., IgA deficiency) could be alpha-gal sensitization [4]. This association seems biologically plausible because the allergen galactose-alpha-1,3-galactose (Gal-alpha1-3Galβ1-(3)4GlcNAc-R) is antigenically similar to the B blood group antigen (Gal-alpha1-3(Fuc-alpha-l,2)Gal). B blood group status offers some protection against development of the allergy. (That is, a self-tolerance to blood antigen B may affect the immune response to alpha-gal.) A possible association of AGS with ABO blood group has been observed in otherwise healthy people: People with group A or O blood may have higher odds of developing AGS compared to the general population and to people with group B blood [5, 6]*.* Due to the increasing incidence of AGS, the potential cross-reactivity of alpha-Gal specific IgE to B type red blood cells poses a possible safety consideration for blood donation and transfusion. We hypothesize that past anaphylactic transfusion reactions may have been associated with alpha-gal sensitization.

Our rationale for choosing to perform a scoping review of the literature on this topic is twofold: first, there is insufficient data in the literature on these specific research questions, due to AGS’s relatively recent emergence. Though several systematic reviews appraise the literature on alpha-gal sensitization [7, 8], these publications did not focus on blood, nor are there major clinical trials for bench-to-bedside treatments for the allergy. A scoping review was therefore deemed suitable for this study. This kind of review was justified over other types of reviews because researchers are now asking broad, exploratory questions about the relationship between alpha-gal sensitization and blood transfusion.

The objective of this study is to perform a scoping review of publications on alpha-gal syndrome and alpha-gal sensitization with no date or language limits to fulfill four research aims:

1. To describe characteristics of all known cases of transfusion-related alpha-Gal syndrome (TRAGS) and hypersensitivity reactions to infusions of mammalian red meat-derived medical products besides blood components that may resemble TRAGS;
2. Identify studies that explore possible relationships between alpha-gal sensitization and blood group that may be relevant to understanding TRAGS;
3. describe which clinical, laboratory, and epidemiologic parameters used to diagnose AGS food allergy are also appropriate to diagnose TRAGS; and
4. Identify what diagnostic assays exist for AGS and how they are used for AGS and/or TRAGS.

## Methods/design

### Protocol and review methodology

The scoping review will follow the methodology described in the *JBI Manual for Evidence Synthesis* [9]. This protocol was prepared in accordance with the JBI methodology for scoping reviews [10]. We will use PRISMA Extension for Scoping Reviews (PRISMA-ScR) as the reporting guideline for the completed review [11].

### Study objectives (research questions)

The research questions are as follows:

1. What are the characteristics of all known cases of transfusion-related alpha-gal syndrome (TRAGS) and hypersensitivity reactions to infusions of mammalian red meat-derived medical products besides blood components that may resemble TRAGS?
2. Do studies show a possible relationship between alpha-gal sensitization and blood group, and is it relevant to understanding TRAGS?
3. Which clinical, laboratory, and epidemiologic parameters used to diagnose AGS food allergy are also appropriate to diagnose TRAGS?
4. What diagnostic assays exist for AGS and how are they used for AGS and/or TRAGS?

### Study team

Our study team includes clinicians and scientists who will screen and extract data for this scoping review in Covidence software. Two clinicians specialized in transfusion medicine and blood banking who have managed cases of possible transfusion-related alpha-gal syndrome [4, 12] will serve as independent reviewers alongside additional reviewers with research focus on tickborne illnesses. Two principal investigators will organize the execution of the study protocol: (1) a principal investigator developing novel diagnostic assays for transfusion-transmitted viruses and (2) a transfusion medicine pathologist and epidemiologist who researches emerging infectious diseases in the U.S. blood supply. Consulting biomedical librarians with extensive experience in performing queries for scoping reviews with biomedical researchers will collaborate with this study team.

### Eligibility criteria

#### Inclusion criteria

This scoping review focuses on alpha-gal and its relation to blood. The population of interest is all persons reported in the literature (pediatric and adult), the exposure of interest is any tick bite or other exposure causing alpha-gal sensitization; the outcomes of interest are evidence of alpha-gal sensitization related to ABO blood group (e.g., epidemiology studies including descriptive statistics, odds ratios, or relative risk calculations) or evidence of allergic or anaphylactic (severe allergic) transfusion reactions associated with alpha-gal sensitization (e.g., case series of transfusion-related alpha-gal syndrome (TRAGS) [13]). We will include published studies (e.g., original research) and grey literature (e.g., clinical trial registries, preprints, abstracts, conference proceedings, posters, evidence-based guidance of health professional societies) with no date limit. Studies published in any non-English language will be reviewed with the assistance of Google Translate.

#### Exclusion criteria

All citations that fail to meet the inclusion criteria will be excluded. There are no other specific exclusion criteria.

### Information sources and search strategy

The following databases will be searched by a biomedical librarian: PubMed (NCBI), which includes MEDLINE; Embase (Embase.com); Cochrane Database of Systematic Reviews and Cochrane Central Register of Controlled Trials (Wiley); Scopus (Elsevier); and Web of Science Core Collection (Clarivate; SCI-EXPANDED [1900–present], SSCI [1900–present], CPCI-S [1990–present], CPCI-SSH [1990–present], BKCI-S [2005–present], BKCI-SSH [2005–present], ESCI [2005–present], CCR-EXPANDED [1985–present], IC [1993–present]) (Supplementary material 1).

Two librarians developed the search strategy with approval and feedback from the study team. An initial limited search of PubMed was undertaken by the librarians to identify key articles on the topic and the text words and index terms contained in the records of relevant articles will be used to develop a full search strategy. The search strategy will contain concepts for red meat allergy, alpha-gal syndrome, and blood, using a combination of standardized indexing terms and keywords (Supplementary material 1) and will be peer reviewed using the PRESS Checklist [10].

The search for grey literature will be carried out in Google Scholar. We will adapt the literature search performed in PubMed to a suitable search in Google Scholar using the search terms ((“alpha-gal” | “alpha-gal”) “blood”). All articles in the first 250 Google Scholar search results will be screened and considered for inclusion. All Google Scholar results following #250 will be excluded, as subsequent results may be irrelevant or duplicative.

The protocol methodology will undergo independent peer review from a qualified biomedical librarian who is experienced with recognized, open access advocating research-funding bodies. They will thoroughly evaluate the study to check that it is scientifically credible and ethically sound in its scope and methods with sufficient detail to instill confidence that the study will be conducted and analyzed properly. The protocol is currently published on Open Science Framework (osf.io) (DOI: 10.17605/OSF.IO/WDZT6) [14].

### Software

The software used for citation management will be EndNote 20 (Clarivate Analytics, London, UK) and Covidence (Melbourne, Australia). Data collection will be performed in Covidence and statistical analyses and data visualization in Microsoft Excel (Redmond, WA, USA) and GraphPad (LaJolla, CA, USA). An image in this protocol was designed in Biorender (Toronto, Ontario, Canada).

### Study selection and screening

**Fig. 1:**
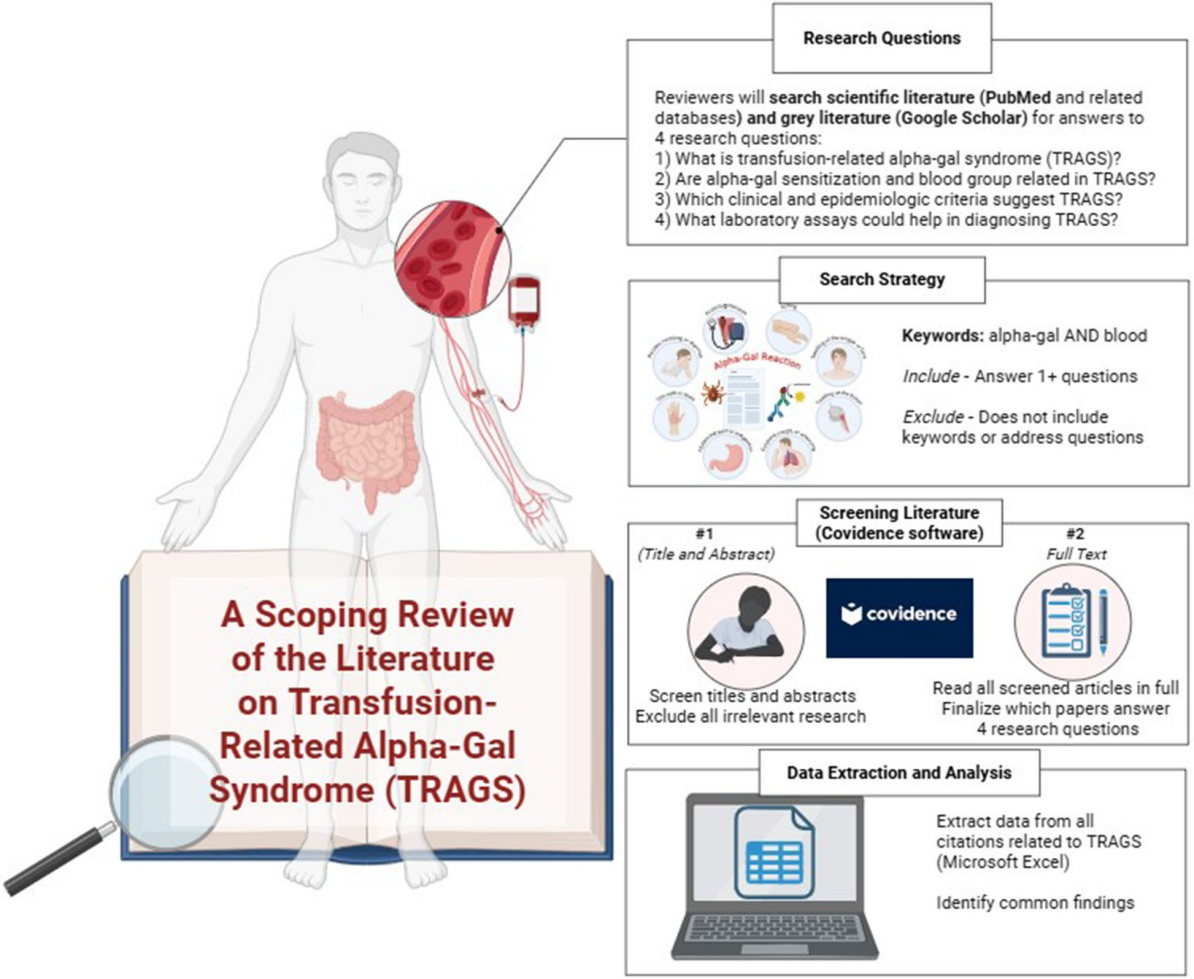
Overview of workflow for the scoping literature review (Biorender)

We will use a two-stage screening process in Covidence (Fig. 1). First, two independent reviewers will screen all titles and abstracts retrieved using the eligibility criteria. If the reviewers find any ambiguous information or disagree about an article’s inclusion or exclusion, an additional reviewer will screen the article and serve as a tiebreaker. The PIs will make any final decisions for inclusion or exclusion and resolve any discrepancies or unclear information. Second, two independent reviewers will screen the full text (abstract, manuscript, tables and figures) of all records included for full text screening in Covidence. If the reviewers find any ambiguous information or disagree about inclusion, the PIs will make the final decision to include or exclude the citation.

#### Study screening pilot

Prior to commencing the scoping review, we will conduct a pilot of all stages of the screening process using a representative sample of 30 articles from the PubMed search selected by the librarians. The articles in the pilot will reflect different kinds of studies (e.g., clinical case series, epidemiologic cohort studies, literature reviews). All reviewers will screen all 30 articles and tag them “Objective #1,” “Objective #2,” “Objective #3,” and/or “Objective #4,” then meet to reach consensus on the classification of all 30 articles (*yes*, article met any one of the four study objectives; *maybe* met inclusion criteria; or, *no*, did not meet inclusion criteria). The study team will then meet with a PI to ensure all reviewers share an understanding of the review process, interpret the eligibility criteria the same way, and handle ambiguous information in a systematic way.

In the full scoping review, inter-rater reliability among all independent reviewers will be compared pairwise to evaluate which pairs of reviewers are more likely to agree or disagree about a citation’s inclusion in the scoping review than would be expected due to chance (Cohen’s kappa statistic) in Covidence software.

### Data collection and data items

#### Data charting form development

We used a pre-existing data charting form before developing a data charting form specific to our scoping review in Covidence. A data collection form developed by health information scientists at Virginia Commonwealth University was used for piloting data collection [15] (Table 1).

**Table 1:**
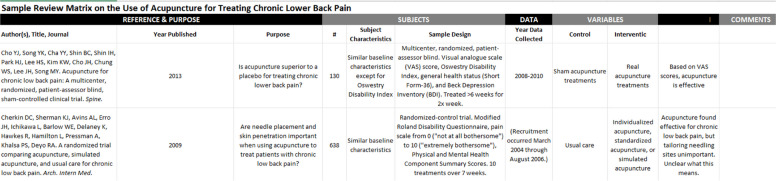
Sample data collection tool used for pilot search and grey literature search (Garrard et al., Virginia Commonwealth University) [15]

#### Data collection

Covidence will be used for data collection of the articles and Microsoft Excel for the grey literature. Two reviewers will collect the data from each included article or grey literature source independently. The PIs will resolve any discrepancies or ambiguous information (e.g., papers that could fall into multiple categories based on relevance to the research questions).

#### Data collection pilot

The PIs will ask at least two reviewers to complete data collection from a single representative article on the research topic (one case series [12], one diagnostic testing study [16], one narrative review [17]) on the previously published data collection form from Virginia Commonwealth University. The PIs will use the reviewers’ feedback on the strengths and weaknesses of the form to create a form containing the data variables most pertinent to comparing the final papers.

#### Data items

In the scoping review, we will extract the following data from the full text of articles that meet inclusion criteria: title; publisher; corresponding author details; geographic area(s) in which the study was conducted; study characteristics (methods, start and end dates of data collection, study funding sources, possible conflicts of interest for study authors); study participants (study population description; inclusion and exclusion criteria; method of recruitment of participants (if human); total number of participants; baseline population characteristics); major results (in abstract or conclusion); conclusion; study strengths and weaknesses; and additional notes with the reviewer’s subjective impression of the quality of the publication:

1. Relevant (discusses alpha-Gal sensitization and blood);
2. Potentially relevant (discusses alpha-Gal sensitization and clinical outcomes that may be tangentially related to blood, or the two topics not in relation to one another);
3. Missing information (discusses alpha-Gal sensitization with no reference to blood);
4. Not relevant (no discussion of alpha-Gal sensitization); or
5. Unknown (all other papers).

The collected data for the grey literature searches will include dates of data entry and collection; full citations for each reference, subjects (subject characteristics and sample design); data (year data collected); variables (control, intervention); conclusion; and the reviewer’s overall impression of the literature.

### Data analysis and synthesis

A PRISMA flow diagram will present the number of studies identified, excluded, and included. We will compile descriptive statistics (counts, percentages) of the number of included studies by (1) article topic (subject area of the journal where the research was originally published) (allergy/immunology, infectious diseases, transfusion or transplantation, other clinical specialties, other laboratory methods, other methods (e.g., library science)) (Table 2) and (2) study objective (Table 3).

**Table 2:** Example of descriptive statistics summarizing data extraction results by article topic

Article topic	Number of references (n, %age)
Allergy/immunology	
Infectious diseases	
Transfusion or transplantation	
Other clinical specialties	
Other laboratory medicine	
Other methods (e.g., library science)

**Table 3:** Example of descriptive statistics summarizing data extraction results by study objective

Study objectives (4)	Number of references (n, %age)
1) Describe characteristics of all known cases of transfusion-related alpha-Gal syndrome (TRAGS) and hypersensitivity reactions to infusions of mammalian red meat-derived medical products besides blood components that may resemble TRAGS	
2) Identify studies that explore possible relationships between alpha-gal sensitization and blood group that may be relevant to understanding TRAGS	
3) Describe which clinical, laboratory, and epidemiologic parameters used to diagnose AGS food allergy are also appropriate to diagnose TRAGS	
4) Identify which diagnostic assays exist for AGS and how they are used for AGS and/or TRAGS	

^*^Note: Some references may fulfill multiple study objectives

## Discussion

### Ethical and safety considerations

This study will involve reviewing previously published literature on predominantly human (and, rarely, animal) studies of diagnostic assays in development for use in humans. The study will not involve clinical research participants, so.

### Dissemination plan

We intend to disseminate the results of our findings to the scientific community, including specialists in allergy and immunology, hematology, and blood banking (e.g., medical directors of blood donor testing programs), and laboratory science, as well as to patients or blood donors who may be experiencing allergic symptoms of alpha-gal sensitization.

### Patient and public involvement

Patients and the public were not directly involved in the development of the scoping review protocol. During the dissemination phase, we may reach out to relevant patient representatives and advocacy groups for people affected by tickborne diseases to ensure that the outcomes of this review are meaningful and useful to those communities.

## Supplementary Information

Supplementary Material 1: Search strategies.

Supplementary Material 2: PRISMA-ScR Checklist.

## Data Availability

The authors reviewed publicly available scientific publications and grey literature and will make their findings available to the public per the most recent policies of the U.S. National Institutes of Health. We intend to disseminate the results of our findings to the scientific community, including specialists in allergy and immunology, infectious diseases, hematology, and blood banking (e.g., medical directors of blood donor testing programs), as well as to patients who may be experiencing allergic symptoms of alpha-gal sensitization. During the dissemination phase we may reach out to relevant patient representatives and advocacy groups for people affected by tickborne diseases to ensure that the outcomes of this review are meaningful and useful to those communities.
